# Development and preliminary validation of a Greek-language outpatient satisfaction questionnaire with principal components and multi-trait analyses

**DOI:** 10.1186/1472-6963-6-66

**Published:** 2006-06-06

**Authors:** Vassilis H Aletras, Efthemis A Papadopoulos, Dimitris A Niakas

**Affiliations:** 1 Business Excellence Laboratory, Department of Business Administration, University of Macedonia, 156 Egnatia st., P.O. Box 1591, 540 06 Thessaloniki, Macedonia, Greece; 2 Faculty of Social Sciences, Hellenic Open University, Patra, Greece

## Abstract

**Background:**

In the recent years there is a growing interest in Greece concerning the measurement of the satisfaction of patients who are visiting the outpatient clinics of National Health System (NHS) general acute hospitals. The aim of this study is therefore to develop a patient satisfaction questionnaire and provide its preliminary validation.

**Methods:**

A questionnaire in Greek has been developed by literature review, researchers' on the spot observation and interviews. Pretesting has been followed by telephone surveys in two short-term general NHS hospitals in Macedonia, Greece. A proportional stratified random sample of 285 subjects and a second random sample of 100 outpatients, drawn on March 2004, have been employed for the analysis. These have resulted in scale creation via Principal Components Analysis and psychometric testing for internal consistency, test-retest and interrater reliability as well as construct validity.

**Results:**

Four summated scales have emerged regarding the pure outpatient component of the patients' visits, namely medical examination, hospital environment, comfort and appointment time. Cronbach's alpha coefficients and Pearson, Spearman and intraclass correlations indicate a high degree of scale reliability and validity. Two other scales -lab appointment time and lab experience- capture the apparently distinct yet complementary visitor experience related to the radiographic and laboratory tests. Psychometric tests are equally promising, however, some discriminant validity differences lack statistical significance.

**Conclusion:**

The instrument appears to be reliable and valid regarding the pure outpatient experience, whereas more research employing larger samples is required in order to establish the apparent psychometric properties of the complementary radiographic and laboratory-testing process, which is only relevant to about 25% of the subjects analysed here.

## Background

Patient satisfaction surveys have been seen as offering an invaluable reflection of the quality of services, especially in countries where other reliable data for quality monitoring is lacking.

The present study focuses on the standard morning operation of the outpatient clinics of Greek National Health System (NHS) hospitals. Any citizen can directly visit any hospital's outpatient facility to see a specialist by merely making an appointment by a telephone call. The service is considered to be complementary to the other existing social insurance schemes of primary care provision. For each visit, the general public, who have social insurance, pay 3 euros only, whereas for public employees and farmers it is free of charge. In fact, 2,565,243 visits have been recorded in 2003 in morning outpatient clinics in the 24 regional and university affiliated NHS hospitals [1].

The need to devise and test a new questionnaire measuring satisfaction, rather than use one of the existing instruments, stems from the idiosyncratic nature of outpatient care in Greek hospitals, together with the lack of appropriate tools adequately tested in this particular setting. The reason we employed a new questionnaire was not that the patients of this country differ significantly from other visitors of outpatient facilities, but rather that the organisation of outpatient clinics varies. Every hospital department offering inpatient care is required at least once a week to provide services, at a rather negligible price, to outpatients who visit these departments at their own free will and without any formal referral from a general practitioner. In each hospital there is an area devoted to the provision of this care, which differs from the facilities of the emergency department. There is also a separate administrative service concerned with the organisation and operation of outpatient care provision. The outpatient clinics aim at the satisfaction of prominent healthcare needs of members of various social security funds (e.g. civil servants, farmers) that do not have their own primary care structures [2].

It is therefore imperative to provide an instrument for measuring patient satisfaction with these outpatient encounters in Greek NHS general short-term hospitals. Towards this end, we devise, pretest and administer a questionnaire and perform reliability and validity analyses to the collected sample data.

## Methods

### Questionnaire development

The items of the questionnaire are the outcome of a literature review, researchers' visits to the hospitals under investigation and a qualitative analysis at the stage of pretesting.

The literature search was conducted using the PubMed database and employing the search terms "outpatient", "satisfaction", "questionnaire" and "hospital". Several outpatient satisfaction surveys resulted, which guided our closed-ended question selection [3-18]. In order for an identified item to be included in our instrument it had to be relevant to the outpatient setting of the particular hospitals under study as well as be appropriate (e.g. not be vague). Questions found in different formats than our satisfaction scale have been reformulated.

The necessary approval for carrying out this study has been provided by the respective Hospital Review Boards of the health care units under investigation. The research team has then closely observed the day-to-day operation of the outpatient clinics. Specifically, three researchers carried out the observations on the most frequently visited clinics, namely cardiology, ophthalmology, orthopaedic, urology, internal medicine, and surgery. Radiographic and laboratory test areas have also been observed. One of the researchers had previously arranged an appointment with the physician at the internal medicine clinic in order to observe the entire process through the patient's eyes [19].

Finally, the qualitative analysis consisted of two rounds of face-to-face interviews each with two head nurses, two physicians, two clerical employees and one hospital administrator. The second round of interviews took place after a first extensive revision of the questionnaire, contained the same number of healthcare professionals and aimed at further refinements of the initial instrument. Healthcare professionals who were asked to participate belonged to different clinics. All the participants were asked to state any deficiencies of the content of the questionnaire, potential other sources of satisfaction and the significance they assigned to individual items. This was meant to ensure content validity.

The questionnaire was then pretested with the use of 12 subjects from different clinics, including males and females, various ages and educational levels. Some of them also had undergone radiographic and laboratory tests, while being in the hospitals. The subjects were presented with the new questionnaire and were asked to express their views regarding the items it contained. Both the concurrent, think aloud and the retrospective methods were used [20]. Moreover, the pretesting process allowed respondents to pinpoint items considered to be important with respect to the satisfaction from the outpatient services, which have therefore been included. The pretesting was conducted three times to a total of 36 different patients, that is, until no additional improvements were considered possible.

The extensive revisions of the early versions of the questionnaire and the pretesting phase increased our confidence that various well-known pitfalls (e.g. biased or vague questions, double negatives etc.) have been avoided.

The instrument should reflect a compromise between its length and completion time and the selection of questions that will adequately represent the experience in the outpatient clinics. The time it takes to complete a questionnaire should not exceed 15–20 minutes in surveys conducted via telephone [19]. Our instrument has a 12–15 minutes completion time in order to ensure acceptability. It consists of closed-ended questions, most of which use a 5-point satisfaction scale (very satisfied, satisfied, neither satisfied nor dissatisfied, dissatisfied, very dissatisfied) accompanied by a "Don't know/Don't wish to answer" option.

The remaining questions are dichotomous, navigation and personal – demographic. The 32 items that measure satisfaction and have been used in the statistical analysis are presented in Table 1. The scale form employed has been seen with some scepticism by some authors [19,21]. Nevertheless, an empirical study that has compared it with the Likert-type and the Expectational scale forms on the basis of their psychometric properties has found generally comparable satisfaction data generated by each scale form as well as comparable measures of reliability [22].

Filter questions ensured that only adults (18+) participated who were not admitted to the hospital as inpatients after their outpatient visit. This decision was due to the fact that admittance might alter the initial impression and satisfaction levels of outpatients and can confound the findings. The questionnaire also included at the end personal information about the participants (age, sex etc.).

### Data collection

The structured questionnaire was administered by means of telephone interviews. In countries where a high response rate can be attained it seems that mail is the preferred mode of administration. Because on-site completion results in biases (that is, favourable responses) since patients feel "prisoners" of the system. Evidence suggests that participants younger than 45 years of age do provide much higher satisfaction ratings on site than they do by mail [23]. There might also be great reluctance to complete the questionnaire and high rates of missing values due to the hastiness to leave the hospital [19]. Moreover, telephone surveys seem to provide more positive ratings than mail-out strategies on personal referent items [24,25]. The magnitude of this social desirability effect varies across studies. One empirical investigation found this effect to be present in more than half of the instrument's items [26], whereas another only in one question [27].

Mail surveys, however, are expected to have a very low response rate in Greece, resulting in significant biases. Perhaps this is why such studies are not customary in this country. One exception supported by the Ministry of Health resulted in a 35.5% response rate [28]. We would expect even lower rates here since in that survey the respective Greek Minister himself signed the cover letter asking citizens to help by stating their views on the quality of NHS hospitals. This problem would be further exacerbated by the lack of detailed annual data necessary to compare sample patient characteristics with those of the hospital outpatient population. We therefore have employed the telephone mode for administering the questionnaire.

Empirical research on optimal call scheduling for telephone surveys suggests that the chances of obtaining an answer and conducting an interview on the first call are significantly better on week-day evenings and on weekends than they are during weekday daytime hours [29]. The time schedule thus included weekdays (18.00–21.30), Saturdays (10.00–14.00 and 18.00–21.00) and Sundays (11.00–13.00 and 18.00–21.00). Calls were not scheduled for holidays. If the respondent was not in at the time of the call or some important activity was interrupted (e.g. dinner), the interviewer would call back another day. Three callbacks have been made before the potential respondent's name was dropped from the list and substituted by another.

Telephone interviews were not computerised. Two interviewers have been used, who were non-professionals. The survey has been administered to the main sample by an independent researcher who had adequate prior experience in public opinion surveys. The second interviewer was a clerical worker with limited prior experience. Both have been trained via studying the questionnaire closely, role-playing an interview, interviewing a supervisor and conducting the interviews in the pretesting phase [19]. A supervisor regularly observed both during this stage and the main survey administration.

We have used stratified proportional sampling of patients booking an appointment on March 2004 to visit the outpatient clinics of a Greek NHS general hospital located in Veroia in Macedonia, Greece. This hospital facility is relatively small in size, having 178 beds in 2003. Stratification was based on sex and specialty (internal medicine, cardiology etc.). The questionnaire was administered within seven days of the visits to the hospital. A second sample was drawn on March 2004 by means of random sampling, with a second measurement to the same subjects occurring within six weeks from the original administration. This sample came from Serres hospital; another NHS institution located in Macedonia, Greece and has been used to assess test-retest reliability, which for technical reasons was not possible in the previous hospital unit. This second unit is bigger having 375 beds. Information necessary to make the telephone calls has been obtained from hospital records.

### Statistical analysis

Most statistical analyses have been performed with SPSS version 12.0 for Windows (SPSS Inc). Nevertheless, the authors themselves have carried out the test for differences in dependent correlations.

In order to reduce random sources of error and be able to assess the reliability and validity of a particular questionnaire it has been suggested to formulate and use summated or multi-item scales [30]. This approach statistically averages out errors related to individual items. The present study employs Principal Components Analysis (PCA) to provide appropriate item groupings [31].

The first step nevertheless in evaluating a questionnaire is to determine the extent of its acceptability by reporting missing data [32]. As such we consider basically the answers of individuals on the "Don't know/Don't wish to answer" option of the questionnaire. Items found to have missing value rates exceeding 10% have been often excluded from further analysis in similar research [11].

Researchers employ various item-component correlation cut-off levels above which an item is incorporated into a summated scale. Values equal to 0.20, 0.40, 0.50 and 0.60 have been previously used [33-36]. We have used 0.50 as the level of acceptance. A second criterion that must be satisfied before an item is included is to load to different components by a difference of more than 0.20, a level suggested by Labarere et al [10]. Note that other authors suggest a more conservative level of differences of 0.30 that was not employed here [35].

The summated scales and the instrument itself have also been assessed by means of various reliability and validity tests [19,30,32]. Internal consistency reliability measures the extent to which all items within a scale are indeed capturing the same construct. Cronbach's alpha coefficients greater than 0.80 indicate high levels of internal consistency, whereas values less than 0.70 suggest that the researcher should attempt to delete individual items from the scales in order to examine whether consistency improves or not [19,37].

We have also assessed the degree to which scales are sensitive to external factors in successive measurements by examining test-retest reliability. An interval between the two measurements of six weeks was employed, following Krowinski and Steiber [19]. As acceptable intraclass coefficients have been considered those with values exceeding 0.50 [38].

Since this was a telephone survey it has been also necessary to examine the consistency of information that has been derived by different interviewers. The questionnaire has been therefore administered twice to a sample of 50 visitors in Veroia hospital by our two interviewers, within a period of one week. The patients have been randomly chosen among the 285 patients that completed the first interview. This short time period was meant to reduce the bias related to the intertemporal stability of scales. Intraclass correlation coefficients must be greater than 0.80 [19]. Interrater reliability has been examined by computing single-measure, two-way random effects, in which each rating was provided by a single interviewer rather than represented an average of many interviewers and, in addition, the latter has been chosen in a random fashion. Moreover, the coefficient was defined in terms of absolute agreement since we were interested in accounting in the analysis of variance the systematic variation from the use of different interviewers [39].

Construct validity assesses the degree to which a summated scale indeed measures the theoretical construct it is designed to measure. This has been attempted with the use of multi-trait analysis [32,33]. According to our methodology, coefficients have been computed to capture the correlation of each item with the scale to which it apparently belongs as well as the correlations of the item with all other summated scales. A high correlation of an item with its own scale (i.e. greater than 0.40) has been taken to indicate convergent validity or internal consistency of the item. A second type of construct validity is called discriminant validity and depicts the degree to which each item within a scale does not measure unrelated constructs. Here we examine whether an item correlates with its own scale more than it does with irrelevant scales.

To further assess construct validity we have also formulated hypotheses that predict some relationships regarding the collected data. In particular, following past research we have expected age to be positively correlated with patient satisfaction [40,41]. There are various explanations for the finding. It could be that older people are more tolerant and stoical than the younger ones; that they engender more respect and care from the hospital staff, or that they have lower expectations due to prior experiences when standards of living were lower. The literature also suggests that perceived health status affects positively the level of patient satisfaction. In fact, it has been observed that patients who have better health report generally higher satisfaction. We have therefore statistically examined this external aspect of validity -often termed "known groups validity" [42]- by reporting correlations of the various scales with the specific characteristics. We have employed in addition to Pearson measures, the Spearman's rank correlation coefficient to account for the possibility of non-normality (skewness) in individual items and the scale scores [41,43]. Since we hypothesised that scale scores would also be correlated to the overall satisfaction level we have also computed correlations for these associations as well.

Finally, we have checked the percentage of respondents at the highest possible (ceiling) or lowest possible (floor) score in the baseline measurement, since these constrain the ability of an instrument to detect changes over time. High ceiling effects might indicate uniform positive attitudes towards hospital services or a failure of included items to capture the full range of values of the constructs.

## Results

### Data collection

From the initial pool of calls, made by the interviewers, we have excluded those that remained unanswered after three attempts as well as those in which the selected responding persons could not complete the interview due to severe hearing problems or inability to speak the Greek language adequately. From the remaining 419 persons that have visited the Veroia hospital and have been called, 285 have agreed to be interviewed and complete the questionnaire, giving a response rate of 68%. From this main sample 60 persons have been drawn randomly and 50 have agreed and completed the questionnaire twice within one week. Sample size has been determined by time and budget constraints rather than the use of formal statistical formulae. Comparable samples, nevertheless, have been used in the literature (sometimes with statistical computation of the necessary sample sizes) even in bigger hospitals [3,8,11]. A second sample of 100 individuals has been drawn from the Serres hospital. From the initial pool of calls we have excluded people that would not meet the criteria for questionnaire completion. The final pool thus consisted of 146 subjects, from whom 107 agreed to participate, yielding a response rate of 73.3%. Another seven have visited the hospital again in the six-week interval under investigation and have been excluded. Hence there were 100 completed questionnaires.

It was not possible to confirm representativness with respect to various demographic – personal characteristics, such as age, educational level etc., due to the lack of the necessary annual population data. Nevertheless, the majority of respondents have been female, middle-aged and elderly, with a low level of education and a good to moderate self-reported health (Table 2).

### Statistical analysis

In Table 3 we see that the "Don't know/Don't wish to answer" reply rates suggest no apparent problems in the understanding of most questions by the respondents. We have decided to retain two items that only marginally reached the 10% cut-off level.

The instrument incorporates inter alia nine questions that measure the satisfaction with the radiographic and laboratory tests. These apply to less than 25% of our sample respondents. Preliminary PCA that included these questions along with all other items gave meaningless groupings of items and low reliability of most scales. It is the case however that the tests are not unique to the outpatient experience. That is, outpatients, inpatients and emergency cases jointly use the relevant hospital resources. The process is somehow distinct and yet contributes to the experience of the visitors of outpatient clinics. We hence also report summated scales and their ratings, but we base these on a separate PCA.

The PCA analysis and components resulting from Varimax rotation are presented in Table 4. Components with eigenvalues less than unity have been ignored. The five remaining components in the first PCA that includes items 1–22 explain 81.42% of the original variance. One component has been dropped since it contained only one item, thus failing to provide a summated scale. In addition, we have only reported items that correlate significantly with a particular component (i.e. with loadings greater than 0.50) as long as they do not also load high on other components (the difference being greater than 0.20). Finally, from the resulting groupings, an item has been deleted if by doing so the respective scale's Cronbach's alpha coefficient improved substantially. The resulting summated scales have been termed "medical examination" (items 15–22), "hospital environment" (items 1, 3, 5, 8, 10), "comfort" (items 7, 9, 14) and "appointment time" (items 2, 4).

In the separate second PCA, which pertains to questions 23–31, the two components explain 74.15 % of the original variance. The scales formed are "lab experience" (items 26–31) and "lab appointment time" (items 24, 25).

The results of the internal consistency reliability analysis are shown in Table 3. All scales have alpha coefficients greater than 0.70. Findings are also encouraging regarding test-retest reliability, given that the respective intraclass correlation coefficients range from 0.74 to 0.96 for the various scales, with very good reliability being implied by values of 0.80 and over [44]. These values are quite high compared to other studies that performed the two measurements within a period of one week to avoid excessive recall bias [41]. Results also indicate high levels of interrater reliability. Coefficients range from 0.89 to 0.99. Finally, the same Table shows evidence for convergent validity with item-total correlations all above 0.40.

Multi-trait analysis is shown in Table 5. We have used the t-statistic for testing the significance of the difference between two dependent correlations from the same sample for all items, as described elsewhere [45]. A sample size of 205 individuals has been used generally, except for computations involving item-scale correlations for the radiographic-laboratory examinations, for which only 43 observations were available. It can be seen that the only scale with an item-scale correlation significantly lower for the hypothesised scale than for competing scales is "hospital environment". Hence we have excluded item 8 of that scale.

Regarding the radiographic and laboratory dimension of the outpatient visit, results are also promising. Nevertheless, an insignificance of many of the differences between correlation coefficients is in fact apparent.

In table 6, it can be seen that age is positively related to the comfort dimension of the outpatient encounter and the laboratory experience aspect, whereas unrelated to other constructs. Better health status is associated with higher levels of satisfaction for all dimensions of the pure outpatient encounter, whereas no association seems to exist concerning the radiographic and laboratory scales. Moreover, as expected, overall satisfaction -as measured by questionnaire item 32- is positively correlated with all summated scales. The highest correlations above 0.65, are observed for the medical examination and the laboratory experience, whereas the lower for the appointment time, for both the outpatient visits and the visits to perform the radiographic and laboratory tests. Floor effect of scales ranges between 0.0 and 4.4%, whereas the ceiling effect varies from 6.3 to 26.3%.

Overall satisfaction with the outpatient services of the hospital located in Veroia (from which our main sample has been drawn) has been measured on a 5-point satisfaction scale. The mean value of item 32 has been transformed into a 0–100 scale and has given a mean overall satisfaction score equal to 75.5. This indicates generally speaking that visitors are satisfied, yet not very satisfied with hospital outpatient services. Ratings are higher for factors relating to the medical examination by the physician (80.53), the environment of the hospital (80.27, without item 8 being included) and the overall experience during the radiographic and laboratory tests (78.41), whilst there is a lower level of satisfaction concerning the comfort of the outpatient experience (70.24), the timing dimension of the appointment booking process for the outpatient clinics (67.82) and the radiographic-laboratory tests (68.35).

## Discussion

In this study we have constructed, pretested and administered an outpatient satisfaction questionnaire employing samples from Greek NHS hospitals. Two separate PCA yielded the initial dimensions of satisfaction for the purely outpatient experience of the visit and the apparently distinct, yet complementary, radiographic and laboratory testing encounter. Higher ceiling effects than the ones observed in this study have often been reported in the literature [46]. Cronbach's alpha coefficients were very satisfactory for most scales, although only marginally so for the "hospital environment" summated scale. Intraclass correlations fully supported the test-retest and interrater reliability of the instrument.

Content validity has been pursued by means of face-to-face interviews with hospital employees and patients during the pretesting stage, which has brought about questionnaire revisions based on their views regarding the significance of various items. Multi-trait analysis and significance tests for differences in dependent correlations suggest that all dimensions of the purely outpatient experience have high discriminant validity. One problematic item was excluded from its respective scale. The radiographic-laboratory dimension seems to have satisfactory validity, nevertheless the lack of significance for the "laboratory experience" scale is widespread and hence highlights the need for a larger sample of patients. This scale should thus be treated with caution.

Although the content of patient satisfaction questionnaires varies greatly across studies and may be context-specific, similar scales have been reported elsewhere. Specifically, one study mentions physician and nursing care as separate scales, whereas our findings incorporated these into the grouping "medical examination" [4]. French research reports "interpersonal skills", "physical surroundings", "convenience" and "appointment delay" that resemble our summated scales "medical examination", "hospital environment", "comfort" and "appointment time" [10]. This latter scale is also found elsewhere [17]. Finally, three of the items in our "hospital environment" category are identical to a similar scale reported in a US outpatient care setting [19].

Regarding the satisfaction of patients from the outpatient services of the hospital located in Veroia, we note that there seems to be more room for improvement in the capability of patients to participate in the scheduling of the appointments to visit the outpatient clinics and laboratory facilities, as well as the waiting time from the days the appointments are booked until the actual days of the visits. There are also lower levels of satisfaction regarding the attractiveness and size of the waiting area outside the physician's office and the availability of seats in order for the patients to wait comfortably for the examination.

Our study has certain limitations. First, the representativeness of the sample patients could not be established since we lacked the necessary population data to perform such statistical comparisons. There is therefore a potential for non-response bias. Moreover, the sample sizes were small, particularly the one used for assessing the reliability and validity of the experience of patients undergoing radiographic and laboratory tests. Finally, the use of samples from many different hospitals would further extend the validation process.

Research regarding satisfaction with outpatient services in Greece has so far been conducted with the use of instruments not assessed for their reliability or validity. One such study identified was conducted on-site in the outpatient clinics of six hospitals located in the southern part of the country [47]. The overall satisfaction from outpatient services was on average 77, on a 0–100 scale. We note that in our study the respective figure was similar (75.5). Of course, the characteristics of the patients in the two samples differ (for instance, our study included proportionally fewer individuals with higher education degrees and younger than 45 years of age).

## Conclusion

Our preliminary validation process has resulted in a questionnaire that measures satisfaction from outpatient clinics and the associated diagnostic procedures. Regarding the pure outpatient component of the hospital encounter, it has yielded summated scales that resemble those found in French hospitals. Despite the similarities, the number and content of incorporated items differ substantially. It should also be noted that one of the scales in that study has had low internal consistency reliability. Psychometric testing shows that our instrument is promising for the measurement of outpatient satisfaction in Greek NHS hospitals. Future research should employ larger datasets from different outpatient populations in order to examine the possibility of generalising the current findings.

## Competing interests

The author(s) declare that they have no competing interests.

## Authors' contributions

VHA has participated in the design and administration of the questionnaire and the study, the literature review, the statistical analysis, the interpretation of the findings and the preparation of the manuscript. EAP has helped in the design of the questionnaire and the collection of the data. DAN has contributed in the design and administration of the survey, the interpretation of the findings and the refinement of the manuscript. All authors have read and approved the final manuscript.

## Pre-publication history

The pre-publication history for this paper can be accessed here:



## Figures and Tables

**Table 1:** Description of questions measuring outpatient satisfaction

Item	Description
1	Ease and promptness of appointment scheduling for the outpatient visit
2	Capability of choosing day and hour for the appointment to visit the outpatient clinics
3	Attitude and conduct of person booking the appointment
4	Waiting time (days waiting) until the visit to the outpatient clinics
5	Outside signage aiding patients to find their way to the outpatient clinics
6	Courtesy and willingness to serve patients at the information desk
7	Attractiveness and size of waiting area
8	Cleanliness of waiting area
9	Ease in finding a seat for pleasant waiting to see a physician
10	Temperature of waiting area
11	Waiting time to be examined by a physician
12	Condition of W.C. (cleanliness, availability of soap, paper etc.)
13	Cleanliness of office in which the medical examination took place
14	Comfort of physician examination office
15	Time devoted by physician for the examination
16	Physician's respect to patient's privacy during the examination
17	Physician's attitude towards patient (i.e. treating him with respect)
18	Willingness of physician to listen to anything the patient had to say
19	Physician's ability to inform patient about his health condition
20	Professional and scientific skilfulness of physician
21	Physician's explanations to patient regarding the suggested treatment
22	Attitude and conduct of other members of hospital staff during the examination (nurses, ancillary staff)
23	Ease of appointment booking for radiographic and laboratory examinations
24	Capability of choosing day and hour for the appointment to perform the radiographic and laboratory examinations
25	Waiting time (days waiting) until the visit to perform the radiographic and laboratory examinations
26	Ease in finding the way to the radiographic and laboratory examination rooms
27	Comfort of radiographic and laboratory examination rooms
28	Radiographic and laboratory staff's attitude and willingness to serve patients
29	Waiting time outside the radiographic and laboratory examination rooms
30	Order with which patients were allowed to be examined by radiographic and laboratory staff
31	Efficiency with which radiographic and laboratory examination was performed (fast, painless examination)
32	Overall satisfaction with the hospital visit to the outpatient clinics

**Table 2:** Characteristics of sample patients

Demographic Variable	"Veroia" Hospital		"Serres" Hospital	
	
	No. of Responses	%	No. of Responses	%
Level of Education				
None	72	25.3	11	11.0
Elementary School	147	51.6	74	74.0
High School – Lyceum	51	17.9	12	12.0
Higher Education	13	4.6	3	3.0
Other	2	0.7	0	0.0
Age				
18–24	4	1.4	3	3.0
25–34	19	6.7	2	2.0
35–44	26	9.2	10	10.0
45–54	42	14.8	14	14.0
55–64	55	19.4	26	26.0
65–74	100	35.2	32	32.0
75 +	38	13.4	13	13.0
Sex				
Male	124	43.5	33	33.0
Female	161	56.5	67	67.0
Health Status				
Good	148	51.9	37	37.0
Moderate	102	35.8	53	53.0
Poor	35	12.3	10	10.0

**Table 3:** Descriptive statistics, internal consistency, test-retest and interrater reliability

Item/Scale		Don't know/wish to answer (%)	Mean	SD	Cronbach's alpha/Item-total correlation†	Test-retest intraclass correlation	Interrater intraclass correlation
Medical Examination (N = 223)*			80.53	15.98	0.96	0.74	0.89
15	Length of examination	6.7	4.11	0.77	0.90		
16	Respect to patient privacy	4.9	3.99	0.88	0.71		
17	Respect – attitude to patient	10.2	4.26	0.66	0.89		
18	Physician's willingness to listen	2.1	4.20	0.73	0.91		
19	Information giving about health	2.1	4.16	0.72	0.94		
20	Physician's skilfulness	6.7	4.20	0.67	0.91		
21	Explanations for treatment	4.9	4.20	0.76	0.92		
22	Attitude of other staff in exam	10.2	4.18	0.68	0.82		
Hospital Environment (N = 268)			79.23	9.31	0.72	0.83	0.93
1	Ease of scheduling appointment	5.7	4.15	0.64	0.51		
3	Attitude of person booking app	4.9	4.28	0.51	0.57		
5	Outside signage	0.7	4.30	0.49	0.49		
8	Cleanliness of waiting area	0	4.01	0.54	0.47		
10	Temperature of waiting area	0	4.10	0.49	0.46		
	Comfort (N = 280)		70.24	15.91	0.80	0.77	0.97
7	Comfortable waiting area	0	3.67	0.81	0.75		
9	Comfortable waiting on a chair	1.1	3.73	0.78	0.73		
14	Comfortable examination room	0.7	4.05	0.64	0.57		
	Appointment Time (N = 270)		67.82	28.90	0.93	0.86	0.99
2	Choice of appointment time	5.3	3.72	1.18			
4	Waiting time for visiting clinic	4.2	3.70	1.22			
	Lab Appointment Time (N = 62)		68.35	25.78	0.87	0.96	0.97
24	Choice of lab appointment time	2.1	3.71	1.08	0.81		
25	Waiting time for visiting labs	1.8	3.76	1.10	0.81		
	Lab Experience (N = 66)		78.41	15.38	0.90	0.94	0.99
26	Ease in finding way to lab rooms	0	4.24	0.69	0.62		
27	Comfort of lab rooms	0	4.04	0.74	0.78		
28	Attitude of lab staff	0.4	4.15	0.63	0.64		
29	Waiting time outside lab rooms	0	3.93	1.01	0.72		
30	Fair queuing system for lab tests	0.4	4.18	0.74	0.71		
31	Efficiency of lab examination	0.4	4.25	0.61	0.70		

*These sample sizes correspond to the main sample from Veroia. Computations on test-retest and interrater reliability have been based on N = 100 and N = 50, respectively.

† These Pearson correlation coefficients have been corrected for item-scale overlap. Correlations for basic scales have been mainly computed for N = 205, that is after deleting missing observations listwise. However, any calculation regarding the radiographic-laboratory scales and items has been based on a smaller sample that was available (N = 43).

**Table 4:** Component loadings*

		Component†			
		
Item	Description	1^st^	2^nd^	3^rd^	4^th^
15	Length of examination	0.898			
16	Respect to patient privacy	0.780			
17	Respect – attitude to patient	0.877			
18	Physician's willingness to listen	0.885			
19	Information giving about health	0.925			
20	Physician's skilfulness	0.894			
21	Explanations for treatment	0.927			
22	Attitude of other staff in exam	0.816			
1	Ease of scheduling appointment		0.685		
3	Attitude of person booking app		0.637		
5	Outside signage		0.808		
8	Cleanliness of waiting area		0.720		
10	Temperature of waiting area		0.635		
7	Comfortable waiting area			0.711	
9	Comfortable waiting on a chair			0.845	
14	Comfortable examination room			0.754	
2	Choice of appointment time				0.753
4	Waiting time for visiting clinic				0.908
24	Choice of lab appointment time		0.894		
25	Waiting time for visiting labs		0.930		
26	Ease in finding way to lab rooms	0.783			
27	Comfort of lab rooms	0.851			
28	Attitude of lab staff	0.803			
29	Waiting time outside lab rooms	0.795			
30	Fair queuing system for lab tests	0.858			
31	Efficiency of lab examination	0.798			

*A separate PCA has been performed for items 23–31.

† Initial components have been derived by PCA. The component matrices have then been rotated with the Varimax method, with the rotation converging after seven and three iterations, for the PCA of items 1–22 and 23–31, respectively.

**Table 5:** Frequency and percentage of item-scale correlations at each level of scaling success*

	-2†		-1		1		2		1+2	
					
Scale	n	%	n	%	n	%	n	%	n	%
Medical Examination	0	0	0	0	0	0	40	100	40	100
Hospital Environment	1	4.0	1	4.0	9	36.0	14	56.0	23	92.0
Comfort	0	0	1	6.6	4	26.6	10	66.6	14	93.3
Appointment Time	0	0	0	0	0	0	10	100	10	100
Laboratory Appointment Time	0	0	0	0	1	10.0	9	90.0	10	100
Laboratory Experience	0	0	1	3.3	16	53.3	13	43.3	29	96.6

* Significance level is set at 5%.

† Levels of scale success:

2: Item-scale correlation is significantly higher for hypothesised scale than for competing scale.

1: Item-scale correlation is higher for hypothesised scale than for competing scale, but not significantly.

-1: Item-scale correlation is lower for hypothesised scale than for competing scale, but not significantly.

-2: Item-scale correlation is significantly lower for hypothesised scale than for competing scale.

**Table 6:** Correlations between scale scores and responses to individual questions*†

Scale/Variable	Medical Examination	Hospital Environment	Comfort	Appointment Time	Laboratory Appointment Time	Laboratory Experience
Age	0.08	0.01	0.22 §	-0.07	-0.11	0.26‡
	(0.08)	(-0.01)	(0.22) §	(-0.07)	(-0.07)	(0.27) ‡
Health Status	0.07	0.19 §	0.07	0.15‡	0.25‡	0.03
	(0.14) ‡	(0.20) §	(0.07) ‡	(0.15) ‡	(0.21)	(0.00)
Overall Satisfaction	0.74 §	0.57§	0.53 §	0.36 §	0.49 §	0.72 §
	(0.65) §	(0.54) §	(0.53) §	(0.33) §	(0.49) §	(0.72) §

* Sample sizes are N = 205 for the pure outpatient experience and N = 60 for items related to the laboratory visit.

† Data outside parentheses are Pearson's correlations, whereas figures inside them represent Spearman's rank correlations.

‡ Correlation is significant at the 0.05 level (2-tailed).

§Correlation is significant at the 0.01 level (2-tailed).
